# A Cure for HIV Infection: “Not in My Lifetime” or “Just Around the Corner”?

**DOI:** 10.20411/pai.v1i1.133

**Published:** 2016-08-16

**Authors:** Michael M. Lederman, Paula M. Cannon, Judith S. Currier, Carl H. June, Hans-Peter Kiem, Daniel R. Kuritzkes, Sharon R. Lewin, David M. Margolis, Joseph M. McCune, John W. Mellors, Timothy W. Schacker, Rafick P. Sekaly, Pablo Tebas, Bruce D. Walker, Daniel C. Douek

**Affiliations:** 1 Case Western Reserve University School of Medicine, Cleveland, Ohio; 2 University of Southern California, Los Angeles, California; 3 University of California, Los Angeles, Los Angeles, California; 4 University of Pennsylvania, Philadelphia, Pennsylvania; 5 Fred Hutchinson Cancer Research Center, Seattle, Washington; 6 Harvard University, Boston, Massachusetts; 7 The Peter Doherty Institute for Infection and Immunity, The University of Melbourne and Royal Melbourne Hospital; Department of Infectious Diseases, Alfred Hospital and Monash University, Melbourne, Australia; 8 University of North Carolina at Chapel Hill, Chapel Hill, North Carolina; 9 University of California, San Francisco, San Francisco, California; 10 University of Pittsburgh, Pittsburgh, Pennsylvania; 11 University of Minnesota, Minneapolis, Minnesota; 12 Harvard University and Massachusetts Institute of Technology, Cambridge, Massachusetts; 13 Vaccine Research Center, National Institutes of Health, Bethesda, Maryland

**Keywords:** HIV, reservoir, eradication, cure, CCR5, functional cure, latency, elite controller, Berlin Patient, inflammation, procoagulant

With the advent and stunning success of combination antiretroviral therapy (ART) to prolong and improve quality of life for persons with HIV infection, HIV research has been afforded the opportunity to pivot towards studies aimed at finding “a cure.” The mere idea that cure of HIV might be possible has energized researchers and the community towards achieving this goal. Funding agencies, both governmental and private, have targeted HIV cure as a high priority; many in the field have responded to these initiatives and the cure research agenda is robust.

In this “salon” two editors of *Pathogens and Immunity*, Michael Lederman and Daniel Douek ask whether curing HIV is a realistic, scalable objective. We start with an overview perspective and have asked a number of prominent HIV researchers to add to the discussion.

**How things have changed:** Once HIV was identified as the causative agent of AIDS, rapid development of diagnostic tests for infection and development of drugs capable of blocking HIV replication shaped the clinical care agenda and altered progressively the landscape of infection. Now, in principle, HIV replication can be contained, allowing preservation or restoration of functional immune competence in most infected persons. Most HIV researchers and clinicians believed that cure of infection was not plausible and, therefore, did not merit further exploration. This perspective was turned around by the remarkable report [1] that an HIV-infected person undergoing treatment for leukemia in Berlin was cured of his infection (and his leukemia) after allogeneic hematopoietic progenitor cell transplantation from a donor homozygous for a defective HIV coreceptor gene—CCR5 Δ32.

Since then, the landscape has changed. HIV cure has become a key priority for AIDS researchers, for both governmental and charitable funders, and for persons living with HIV infection. Among all this optimism, a variety of radically different approaches have been proposed to eliminate infection; yet, and although the field is very young, the “Berlin Patient” remains the only person to have been cured. Notably, a small number of cases that have attempted to replicate that result have failed [2].

**Complete HIV eradication will not be simple and may not even be possible:** The barriers to complete HIV elimination are substantial. Virus quiescence in host cell genomes renders infected cells not readily visible to host defenses; moreover the frequency of HIV-specific CD8 T cells typically decreases as HIV antigen levels fall with ART and these cells often have an “exhausted” (or dysfunctional) phenotype [3, 4]. Infected cells are broadly distributed in numerous tissues, including sites that may be relatively inaccessible to host defenses or treatment strategies [5], and the limits of sampling render our best current assays for residual infectious virus incompletely sensitive to detection of viral persistence in these tissues [6]. Recognizing that complete eradication is a daunting objective that may not be readily scalable, affordable, or possible, some treatment strategies are proposing a less definitive outcome that has come to be called **“functional cure”**—broadly defined as a state in which ART may be withdrawn without subsequent virus recrudescence. The concept is akin to that of “complete remission” after treatment for malignant cancer.

**Natural control of HIV infection:** The plausibility of functional cure has been guided by the demonstration that certain rare HIV-infected persons, “elite controllers” can control viremia in the absence of antiretroviral therapy [7, 8]. More recently, studies of participants in the French Visconti cohort have suggested that some persons in whom ART is initiated early in the course of infection can durably interrupt ART while sustaining virologic control [9]. While strategies capable of achieving this clinical phenotype can teach us a great deal about the determinants of host defense against HIV replication, this outcome, as currently understood, may not be as clinically beneficial as those that are already achievable with ART.

**Disease progression in elite controllers:** While the elite controller state is associated with sustained low levels of plasma viremia in the absence of antiretroviral therapy, indices of inflammation and coagulation are higher than among persons in whom HIV replication is controlled by ART [10]. Moreover, while progressive CD4 decline is rare in ART-controlled HIV infection (Benigno Rodriguez, personal communication, August 2016), it does occur among elite controllers [11]. In part for these reasons, the AIDS Clinical Trials Group has launched a controlled clinical trial of ART in elite controllers to ascertain whether treatment affects the inflammatory and CD4 T cell trajectory in these persons.

With these issues in mind, is the goal of achieving functional cure as defined by elite controller status good enough? While this may not be a clinically desirable ultimate objective, strategies that result in “functional cure” could plausibly contribute to a multi-component regimen that might eradicate infection. Perhaps we should regard functional cure as an experimental target that may help to reveal the determinants of host control of HIV replication and its persistence. Treatment strategies designed to achieve a functional cure should include careful monitoring of inflammatory and procoagulant indices and, if these remain elevated, serve as a warning that the strategy alone may not be optimal. Nonetheless, strategies that induce control of HIV replication or that decrease the size of the HIV reservoir(s) could plausibly be part of more comprehensive approaches designed to completely eliminate (cure) HIV infection. Testing these strategies could clarify the roles of the mechanisms induced or targeted in HIV replication and persistence and, as such, have intrinsic value; whether they will alone confer clinical benefit comparable to or exceeding that achieved by ART is another question entirely.

## CHALLENGES TO “THE CURE”

**Where does HIV persist?** While the latently-infected CD4 T cell is the predominant “reservoir” of cells containing infectious virus, there may be others (such as macrophages), and infected cells of any type may be distributed in anatomical sites less reliably accessible to antiretroviral drugs [5]. These reservoirs need to be better understood, with the goal of defining how many potentially infectious viruses are present in an infected person whose viremia is “fully suppressed” by ART. We already recognize from experience with hematopoietic progenitor cell transplantation that virus can persist and reactivate despite levels in peripheral blood that escape our assay detection limits [6]. A single cell with an intact provirus may be enough to “ruin” an attempt at a cure.

**Quiescence** is also a challenge to host immune defenses. Ongoing HIV replication promotes virus persistence through selection for resistance and escape from host defenses. Suppressive antiretroviral therapy will halt this evolutionary mechanism for virus survival, but the persistence of infectious virus genomes that are not expressing virus proteins allows these infected cells to serve as durable reservoirs of infectious virus that remain indifferent to any host defense that targets expressed virus elements.

**Targeting the HIV reservoir:** Many novel strategies are being explored in an effort to completely eradicate HIV infection. The majority will likely fail and, for the reasons outlined above, even those that reliably decrease the size of the HIV reservoir may be insufficient to cure HIV. We still do not have a sense that there exists a level of reservoir reduction—without complete elimination—that would suffice to result in durable control of HIV as defined by the absence of viremia. In this regard, the limits of our ability to monitor virus persistence make it impossible to know whether every infectious virus has been eliminated from the one person who has been cured. In the Berlin Patient, replacement of the entire immune cell pool with cells resistant to HIV likely rendered the emergence from latency of any residual infectious virus incapable of amplification. It remains to be seen whether HIV can be eradicated from any person without also rendering all or most of the host cellular targets for infection “resistant” to HIV.

**Can the host be made resistant to HIV?** A number of strategies are being explored that include introduction of resistance elements or gene editing to render immune cells resistant to HIV infection [12]. As noted, replacement of host immune cells with “naturally” HIV resistant allogeneic cells likely was a major determinant that led to the cure of the Berlin Patient. And while CCR5-deficient cells were apparently sufficiently resistant to infection with the viruses infecting this patient, it is not clear that this approach will be effective in all individuals, some of whom may harbor viruses capable of binding to other coreceptors, such as CXCR4 or others [13]. Thus, for some (or many), other resistance strategies will be necessary. Altering the nature of target host cells to render them HIV-resistant might work, but it remains to be seen whether a scalable strategy can be devised that can safely destroy and purge enough latently infected cells and safely replace enough of the HIV-susceptible target cell with resistant cells. Unknowns here include how much purging and how much replacement will be necessary to result in durable control of HIV replication.

**Will HIV cure normalize the perturbed immune homeostasis and the inflammatory/procoagulant state of treated HIV infection?** As noted above, many persons with ART-suppressed infection maintain low circulating CD4 T cell numbers, increased circulating CD8 T cell counts, and an increased proinflammatory and procoagulant state that have been linked to ART-era morbidity and mortality [14–16]. Setting aside for the moment whatever costs there might be to the host of any plausible cure strategies, we should ask what would happen to these immune, inflammatory, and coagulation abnormalities if all remaining HIV could be made to disappear? This “thought experiment” isn't completely fanciful, as we must take into consideration the long-lasting effects that sustained periods of HIV replication and its consequences have had on immune and inflammatory homeostasis. Though the drivers and the mechanisms of these perturbations are incompletely understood, persistent fibrosis and immune dysregulation are readily demonstrable in gut and lymphoid tissues in treated HIV infection and are linked to the immune restoration failure [17] and persistent systemic inflammation [18] that are both linked to ART-era morbidity [14, 19]. Because it remains unclear whether these architectural residua of chronic infection will resolve with HIV eradication, we should be prepared to consider the possibility that they will not—and explore strategies to correct them.

**Do we need a cure?** Early ART was poorly tolerated, complicated, and far less effective than current combination regimens. These days, many persons with HIV infection can achieve durable control of HIV replication with once daily administration of well-tolerated single pill combination agents. Furthermore, those who start treatment early are likely to sustain normal circulating CD4 T cell numbers, remain free of opportunistic infections, and have a predicted survival similar to that of uninfected persons [20]. Thus, the bar for a scalable cure strategy is high!

**Community engagement:** There is great community interest in HIV eradication and clinical studies that target HIV eradication are particularly attractive to many persons infected with HIV. It is important that these clinical trials don't promise too much, and that they provide a realistic perspective on risks and plausible outcomes.

**Summary:** There are numerous scientific, technical, and logistical challenges to curing HIV infection and the HIV research community is poised to address them. Will any of these strategies succeed in providing a scalable approach to HIV eradication in our lifetime? This remains to be seen, and how exactly does one define a lifetime? There are already an estimated 37 million HIV-infected persons worldwide who are in need of lifelong treatment. Now a key goal remains to deliver treatment to all who need it. And while there are formidable challenges to HIV eradication, there is much to be learned from cure trials that are creatively and thoughtfully designed.

## COMMENTARIES

### Paula Cannon

HIV infection is a failure of the immune response, even when supported by the most effective ART. Like a cancer escaping chemotherapy, it reflects the limitations of what we can do against this most insidious of human pathogens. But recent advances in cancer treatments have shown the potential of a smart(er) immune response and for HIV we can do the same. Gene therapy is providing the tools to act on the explosion of knowledge of immune responses that HIV research has itself contributed. We can manipulate cells to be both HIV resistant and more HIV responsive. It is time to make these tweaks and stand back and let the immune system do the heavy lifting.

### Judith Currier

Despite the significant challenges that lie ahead in the attempts to cure HIV, the path is worth pursuing for several reasons. First, if there is a cure down the road, it will likely work best for those who start treatment early. This adds yet another potential incentive for starting therapy as early as possible for those diagnosed with HIV. If more people start treatment early that will help to slow the epidemic and everyone benefits. Second, research towards a cure that includes careful study of post-treatment controllers could shed light on key immunologic responses that can inform vaccine research. And finally, despite the improvements in therapy available in some parts of the world, many people living with HIV still hope that one day they will be free of the virus. Having one person cured is a strong gravitational force to keep us going down this path.

### Carl June

I am not optimistic that immune and vaccine approaches will be sufficient for “cure” strategies, and that in cases where an “elite controller” status can be successfully induced, it will come at an unsustainable fitness cost to health in the form of inflammation and accelerated aging. On the other hand, I am optimistic that installing an HIV-resistant immune system using the principles of synthetic biology is possible. I am most optimistic regarding approaches that utilize engineered HIV-resistant T cells and stem cells to phenocopy a graft vs leukemia effect (i.e., to purge endogenous CD4 T cells), and to use cellular host restriction factors to block infection. We have many new tools for genetic editing and cell engineering to accomplish the above.

### Hans Peter Kiem

I think there will be a cure for HIV but, just as with cancer, there will likely not be one magic treatment that will be a cure for all HIV patients. I believe 2 important issues must be addressed to facilitate HIV cure research. **1) Definition of HIV cure:** Measuring complete HIV eradication is very difficult and may be impossible to accomplish in a timely fashion. When treating cancer, we talk about remission and long-term remission and then eventually about cure, a process that takes many, many years and in most cases at least 5 years of complete remission. However, functional cure, that is, “a state in which ART may be withdrawn without subsequent virus recrudescence” can easily be measured and studied and would allow us to evaluate novel, promising treatment strategies very quickly and efficiently, and we may be able to identify situations and mechanisms that lead to HIV eradication. **2) Taking advantage of novel immunotherapies:** The recent enormous advances in using gene therapy/editing for immunotherapy in the cancer field to target specific cancer antigens will allow us to use this technology to target specific HIV antigens and combine this approach with other promising treatments such as the use of broadly neutralizing antibodies or vaccines. In addition, we can use the gene editing technology to render our immune system resistant to HIV infection, which will further boost the patient's immune system to fight and control HIV infection.

### Daniel Kuritzkes

There is no guarantee that even if cure is achieved it will lessen the long-term consequences of having once been HIV infected, or that the inflammatory state will return to baseline. This remains an untested hypothesis, and an important reality check.

Will approaches to cure be “scalable”? Clearly, this is an important issue for general implementation, but not essential for proof of concept. If an approach to cure can be found that is less drastic than what was applied in the case of the Berlin Patient, one could then work on refinements to make it scalable. Azidothymidine (AZT) administered Q4H was not really scalable, but we've moved on from there.

Do we need a cure? First we must acknowledge that whereas current treatment is highly effective, safe, and convenient, it nevertheless requires lifelong administration and reasonably strict adherence. The question of the sustainability of providing lifelong ART to the 37 million in need is a worry. In addition, patients speak of the stigma of HIV infection, and the stigma associated with the need to take ART. I fully agree that community expectations must be managed realistically and we need to be careful not to over-promise; nevertheless, to some extent the groundswell of enthusiasm around cure research on the part of patients is motivated at least in part by the stigma issue, and in part by unrealistic expectations about the state of health to which they might be restored absent HIV.

### Sharon Lewin

In the last 5 years, considerable progress has been made in understanding where HIV persists, developing new methods to measure virus persistence, identifying relevant animal models, and defining what will be needed for an effective intervention. It is now clear that simply reducing persistent virus will not be sufficient. Long-term immune surveillance will also be critical to ensure that rebound virus doesn't occur. I believe both strategies will be needed and both present unique challenges. Although treatment is now relatively simple and increasingly cheap, the global burden of maintaining lifelong treatment for all who need it, especially in low income countries, is formidable with respect to both cost and health infrastructure. Therefore, there is a high need for a continued concerted collaborative effort to find a way to safely stop ART and to ensure that the end product will be cheap, scalable, and available globally.

### David Margolis

“It is our task, both in science and in society at large, to prove the conventional wisdom wrong and to make our unpredictable dreams come true.” —Freeman Dyson

The many unknowns and challenges of research towards HIV remission or cure are well outlined here. Although it would not address the desires of most living with HIV infection, the argument could be made that the tools to eventually end AIDS by completely interrupting ongoing transmission are already at hand. However, the true metrics of the events that drive persistent HIV infection or thwart its clearance—the lifespan of latently infected cells, the frequency with which new latent infections arise during suppressive therapy, the true efficiency of serial latency reversal therapy *in vivo*, and the effectiveness of natural or engineered antiviral clearance mechanisms—have not been directly and precisely measured. Therefore it is impossible to know whether a cure is one breakthrough away or requires several decades of determined effort to be realized.

### Mike McCune

From the hallways of San Francisco General Hospital, reflecting on three-plus decades in the midst of the HIV epidemic, recalling the time when very few survived infection, I find it both remarkable and inspiring to read a review such as this. Not only has the life expectancy of HIV-infected individuals been extended from weeks to many years, the community is now envisioning a time—perhaps far off but nonetheless in focus—when there might be a safe, scalable, and affordable cure. Douek and Lederman discuss the allure of such a cure while also honestly revealing the barriers ahead. Such balance should help us to walk forward with purpose during the upcoming years—and to thoroughly test the possibility that a cure is possible. Whatever transpires, it is wonderful to know that this effort is being tackled with such great thought and drive.

### John Mellors

The remarkable success of current ART, which largely consists of a single tablet taken daily with minimal to no adverse effects, makes achieving sustained remission of HIV without ART a very daunting challenge. The safety and efficacy of current ART also lowers the tolerance that the scientific, regulatory, and patient communities will have for adverse consequences of therapies designed to achieve an HIV cure. For these reasons, I believe the best opportunity to achieve a safe HIV remission is through the use of “designer” monoclonal antibodies that are generally very safe and that promote the clearance of HIV-infected cells through immune effector cells (phagocytes and NK cells) combined with therapeutic vaccines that induce broad, polyfunctional immune responses to highly conserved HIV epitopes, keeping virus in check that emerges from latency. Other approaches including latency reversing agents, gene therapy, autologous hematopoietic transplantation, cellular therapy with modified T-cell receptors (e.g., CARs) and immune checkpoint inhibition (e.g. anti-PD-1/PD-L1) all have higher inherent risks of toxicity, many of which may prove to be unacceptable in the context of currently safe, convenient, and very effective ART. It is also important to consider that the efficacy of any curative approach is likely to vary by the stage of HIV disease at which ART was started. If ART had been delayed until severe immunodeficiency was present, it is likely to be much more difficult to repair immune dysfunction and generate broad and effective HIV-specific immune responses than if ART had been started sooner after infection when immune damage was minimal. Early initiation of ART also has the major benefit of stopping HIV replication and evolution before a swarm of immune escape variants can emerge. Restricting the size and diversity of HIV reservoirs will reduce the number of viral variants that need to be controlled by antibody therapy and/or vaccination. For these reasons, if sustained remission of HIV can be achieved, it is most likely to occur first in individuals treated with ART at the earliest stages of infection, although this group represents a small proportion of individuals living with HIV infection.

### Timothy Schacker

HIV infected people on therapy are not immunologically normal, continue to have significant medical problems, and have early mortality. We know a cure is possible if we can find a way to recapitulate the steps that led to a cure in the Berlin Patient (first ablate the reservoir and then prevent its reestablishment) in a way that is safe and economically feasible. Immunotherapy for cancer is providing remarkable results in conditions that were otherwise lethal and may provide a strategy to eliminate virus-infected cells, thus ablating the reservoir. The field of gene therapy is advancing quickly and there are already clinical trials in HIV infection to provide cells that can't get infected with HIV. These remarkable advances make me believe a cure is possible, but we should never lose sight of the fact that most people with the infection live in resource-limited settings where delivery of complex medical therapies is, at best, challenging. Having said that, I firmly believe we should be exploring all of these exciting opportunities, as I think it very likely they will eventually lead us to at least a functional, if not complete, cure.

### Rafick Sekaly

Immune homeostasis, the process that leads to the maintenance of memory T cells and, by inference, the HIV reservoir is under the control of regulatory T cells and the cytokines they produce: TGF beta and IL-10. These cytokines are triggered in response to the proinflammatory environment that results from virus sensing by pattern recognition receptors and by bacterial translocation. They disrupt homeostasis by creating an environment of immune quiescence and immune senescence, which prevents the replacement of latently infected cells by new cells. This establishes the HIV reservoir. Resetting homeostasis through interventions that are less invasive than those that were administered to the Berlin Patient will help eradicate HIV. Controlling Treg function could be an easier fix than once thought.

### Pablo Tebas

Timothy Ray Brown [The Berlin Patient] proved that HIV can be eradicated in the same way that Neil Armstrong proved that humans can go to the moon. Sometimes in science you only need n=1 experiments. I think we will reach first a functional cure: patients will not have to take antiretrovirals and will not have ongoing issues with HIV. I do not know if it will be with a therapeutic vaccine, activators or inhibitors of the reservoir, immunomodulators, gene therapy, or a combination of these. Curing HIV will not be easy and might not happen often, but is it worth trying? Absolutely, because, to rephrase Judy Garland, “The dreams that we do not dare to dream really do not come true.”

### Bruce Walker

The answer to whether we can cure HIV infection has to be yes. A single patient has proven this—with the caveat that ongoing follow-up is still needed. If current paradigms hold, cure is not likely to happen soon, and scalability is an even greater reach. A major question is how to speed the discovery process that is likely to be required to reliably achieve cure. Studying elite controllers and post-treatment controllers may provide insights, but a similar functional cure is unlikely to be a satisfactory outcome. Interventional studies in persons who are treated early in acute infection may represent a lower bar due to smaller reservoir size. Immune augmentation with vaccines or administration of broadly neutralizing antibodies may provide access to germinal center reservoirs, which may be the main drivers of persistence. The bottom line is, we still have a lot to learn.
